# Comparative Analysis of Fecal Microbiota Composition Between Rheumatoid Arthritis and Osteoarthritis Patients

**DOI:** 10.3390/genes10100748

**Published:** 2019-09-25

**Authors:** Jin-Young Lee, Mohamed Mannaa, Yunkyung Kim, Jehun Kim, Geun-Tae Kim, Young-Su Seo

**Affiliations:** 1 Department of Internal Medicine, Kosin University College of Medicine, Busan 49104, Korea; rejim@hanmail.net (J.-Y.L.); efmsungmo@hanmail.net (Y.K.); libertier@gmail.com (J.K.); 2 Department of Integrated Biological Science, Pusan National University, Busan 46241, Korea; mannaa_mohamed@yahoo.com

**Keywords:** rheumatoid arthritis, osteoarthritis, gut microbiota

## Abstract

The aim of this study was to investigate differences between the gut microbiota composition in patients with rheumatoid arthritis (RA) and those with osteoarthritis (OA). Stool samples from nine RA patients and nine OA patients were collected, and DNA was extracted. The gut microbiome was assessed using 16S rRNA gene amplicon sequencing. The structures and differences in the gut microbiome between RA and OA were analyzed. The analysis of diversity revealed no differences in the complexity of samples. The RA group had a lower Bacteroidetes: Firmicutes ratio than did the OA group. Lactobacilli and *Prevotella*, particularly *Prevotella copri*, were more abundant in the RA than in the OA group, although these differences were not statistically significant. The relative abundance of Bacteroides and *Bifidobacterium* was lower in the RA group. At the species level, the abundance of certain bacterial species was significantly lower in the RA group, such as *Fusicatenibacter saccharivorans, Dialister invisus, Clostridium leptum, Ruthenibacterium lactatiformans, Anaerotruncus colihominis, Bacteroides faecichinchillae, Harryflintia acetispora, Bacteroides acidifaciens, and Christensenella minuta*. The microbial properties of the gut differed between RA and OA patients, and the RA dysbiosis revealed results similar to those of other autoimmune diseases, suggesting that a specific gut microbiota pattern is related to autoimmunity.

## 1. Introduction

The human gut is one of the richest microbial ecosystems, containing more than 5000 different species with wide variation in their genetics and biochemistry. The gut microbiota start building up at birth and several environmental and host-related factors are involved in shaping the established microbial composition [1,2,3]. Gut microbes have numerous direct and indirect effects on human health, as they have roles in host cell development, nutrition, and immunity [4].

Rheumatoid arthritis (RA) is a very common systemic autoimmune disease that leads to progressive joint destruction [5]. So far, RA has been the main focus for studying the microbiome’s role in autoimmunity [6]. Multifactorial etiological factors could be involved in autoimmune diseases, and the role of gut microbiota is being more appreciated with recent studies [7,8]. A change in microbiome ‘dysbiosis’ was found to be involved in several diseases, including inflammatory bowel disease, obesity, diabetes mellitus, autism spectrum disorder, and cancer. It could also be strongly linked to autoimmune disease in people with certain genetic backgrounds and environmental factors [9]. In addition, specific gut microbial community structures have been found to be important determinants of disease onset in RA patients [10,11].

Osteoarthritis (OA), the most common type of arthritis, is a degenerative disease, and the role of inflammatory factors, such as those from microbiota, has been considered minor [12]. The exact etiology of OA is not yet known, but it has been recently linked to individual-specific factors like age, sex, obesity, and diet [13,14]. Aspden et al. [15] reported that the intestinal microbiota is an important hidden risk factor for OA and it may be an important indicator of individual-specific risk factors. It has become clear that the activation of inflammation in obese patients is caused by the alteration of the intestinal microbiome [16,17].

There have been reports of several mechanisms by which changes in the intestinal microbiota can cause joint disease. First, nutritional absorption in the intestine is regulated to some extent by the intestinal microbiome [18]. The intestinal symbiotic flora is the source of several vitamins that may affect the whole body and the musculoskeletal system [19]. Second, the immune cell response to the intestinal flora may lead to the secretion of pro- and anti-inflammatory components into the systemic circulation, from where they eventually travel to the bone and the synovium. The translocation of microbes and microbial metabolites across the gut endothelium is another process by which the microbiota may influence bone and joint disease. While the gut lining forms a barrier that limits transport across the endothelium, microbial-associated molecular patterns (MAMPs) often penetrate the gut endothelium and enter the systemic circulation [20].

While there is a considerable amount of research on RA links to gut microbiome, the gut microbiome in patients with RA in comparison with that in OA patients requires further studies. In this study, we attempted to distinguish RA and OA by comparing the fecal microbiome and describing its potential association with both diseases using a non-invasive strategy.

## 2. Patients and Methods 

### 2.1. Patients

This study was approved by the Institutional Review Board of Kosin University Gospel Hospital (2017-08-15). A total of 18 Korean patients were recruited at the outpatient Division of Rheumatology and were divided into two groups, RA (*n* = 9) and OA (*n* = 9), based on the diagnostic criteria. RA patients were confirmed according to the classification criteria in the American College of Rheumatology (ACR)/European League Against Rheumatism (EULAR) 2010; the patients were also naïve to biological drugs. The disease duration, activity markers, and medication use among the RA patients are shown in Table S1. OA patients met the 1995 revised ACR classification criteria. Any patient who was being treated with antibiotics, consuming probiotics, or had a history of inflammatory bowel disease or other autoimmune diseases was excluded from the study. Written informed consent was obtained from all study participants. Erythrocyte sedimentation rate (ESR) and C-reactive protein (CRP) were assessed on the day of stool sample collection. 

### 2.2. Sampling and DNA Extraction

Fresh fecal samples from study participants were collected in sterile containers and immediately stored at −80 °C until further processing. Total metagenomic DNA in 250 mg (fresh weight) of the homogenized fecal samples was extracted using PowerSoil^®^ DNA Isolation Kit (MO BIO Laboratories, Carlsbad, CA, USA), following the manufacturer’s protocol. The obtained DNA were checked for concentration and quality using the NanoDrop2000 spectrophotometer (Thermo Fisher Scientific, Wilmington, CA, USA) and by agarose gel electrophoresis (0.8% agarose in Tris-borate-EDTA). The qualitatively assessed samples of intact metagenomics DNA with appropriate A260/A280 and A260/A230 ratios were stored at −20 °C in TE buffer until they were used in sample preparation, 16S rRNA amplification, sequencing, and sequence analyses.

### 2.3. Sequencing, Bioinformatic Analysis, and Assignment of the Microbiota Composition

The V3 and V4 variables of the 16S rRNA region were amplified and the sequence library was generated by Polymerase chain reaction (PCR) using the following primer pair:

(F), 5’-TCGTCGGCAGCGTCAGATGTGTATAAGAGACAGCCTACGGGNGGCWGCAG

(R), 5’-GTCTCGTGGGCTCGGAGATGTGTATAAGAGACAGGACTACHVGGGTATCTAATCC.

The amplification, sequencing, and library preparation workflow were conducted using Herculase II fusion DNA polymerase Nextera XT Index Kit V2, following the 16S metagenomic sequencing library preparation Part # 15044223 Rev. B protocol in Illumina^®^ MiSeq^®^ platform at Macrogen (Seoul, South Korea) yielding paired-end reads. Paired-end reads were merged using Fast Length Adjustment of Short reads (FLASH; http://ccb.jhu.edu/software/FLASH/) [21]. The taxonomic assignment and diversity statistics were performed using the Quantitative Insights Into Microbial Ecology (QIIME) (company, city, country) pipeline for demultiplexing, taxonomic assignment, and diversity statistics from the phylum to the species level [22]. The generated raw data were first quality trimmed and purified using Scythe (v0.994) and Sickle programs to remove adapter sequences and short and low-quality reads. The CD-HIT-OTU-MiSeq approach and UCLUST were used for the clustering and annotation of the qualified processed non-chimeric sequences into the respective Operational Taxonomic Units (OTUs) using a greedy algorithm at 97% cutoff against the Greengenes database [23,24]. The obtained sequences were deposited to the GenBank under sequence read archive (SRA) accession number PRJNA511932.

### 2.4. Statistical Analysis

Statistical analysis and data plotting for alpha- and beta-diversity were performed using QIIME scripts and R (version 3.1.3). The stacked bar graphs were used to show the relative abundance of different bacterial taxa for each individual. The means ± standard deviations of the differentially existed taxa between the RA and OA groups were statistically analyzed using two-tailed, independent samples *t*-test, where a *p*-value < 0.05 was considered to be statistically significant.

## 3. Results

### 3.1. Characteristics of the Study Participants and the Obtained Reads

The study participants of both groups, RA and OA, were similar in their age, body weight, and body mass index. However, RA patients had significantly higher levels of the ESR and hsCRP (Table 1). Rheumatoid arthritis patients were all females, while those with osteoarthritis were 8 females and 1 male. The number of patients with diabetes mellitus (DM) was both 1 (11.1%) in RA and OA. And the number of patients with hypertension was both 2 (22.2%) in RA and OA. The characteristics of RA patients are listed in Table S1. The disease duration was more than 1 year and the disease activities (DAS28-ESR) were low-to-moderate in range. Antirheumatic drugs were mostly used in combination, rather than alone. Six patients had seropositive rheumatoid arthritis and the other three had seronegative rheumatoid arthritis.

**Figure 1:**
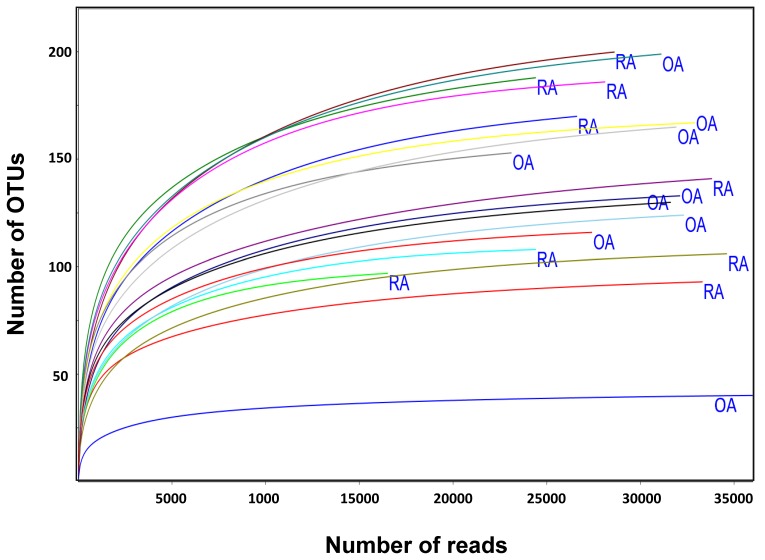
Rarefaction curves of the numbers of reads and the number of assigned phylotypes from the fecal sample of rheumatoid arthritis (RA) and osteoarthritis (OA) patients.

There was no significant difference in the number of qualified reads between RA (27,955 ± 5761) and OA (32,430 ± 6698) patients. The rarefaction curves of the number of reads plotted against the number of OTUs indicated that the number of qualified reads from the used samples were adequate for the final analysis, as a further increase in the reads would have a minor effect on the obtained OTUs (Figure 1).

### 3.2. Diversity Analysis of the Fecal Microbial Composition Between RA and OA Patients

**Figure 2:**
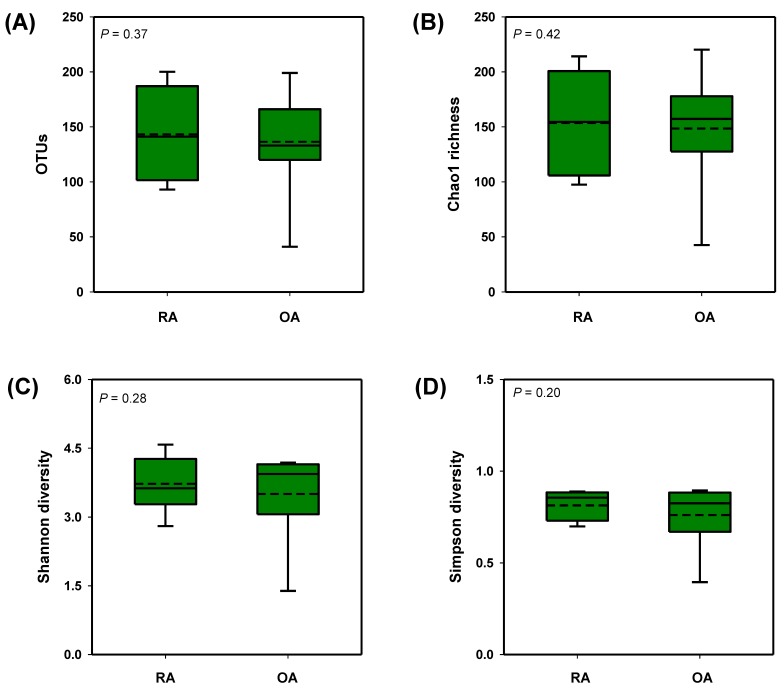
Box plot representing (**A**) the OTUs, (**B**) Chao1 richness, and (**C**) Shannon Diversity and (**D**) Simpson diversity Indices from the fecal sample of rheumatoid arthritis (RA) and osteoarthritis (OA) patients. Means and medians (*n* = 9 for each group) are shown with straight and dashed lines, respectively, inside the box; *p*-values shown on top left side of each graph were based on independent samples *t*-test.

**Figure 3:**
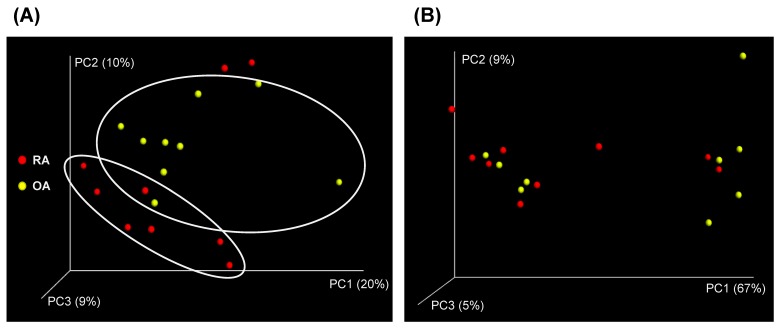
Three-dimensional diagram of the principal coordinate analysis (PCoA) of (**A**) unweighted UniFrac and (**B**) weighted UniFrac distance metrics of the OTUs abundance data from the fecal sample of rheumatoid arthritis (RA) and osteoarthritis (OA) patients.

The diversity analysis revealed that there were no apparent differences in the complexity within samples represented as OTU numbers and the alpha-diversity, Chao1 richness, and Shannon and Simpson indices between the two groups (Figure 2). However, there was a moderate divergence observed between the RA and OA fecal microbiota as shown in the three-dimensional (3D) graph of the unweighted (qualitative) UniFrac principle coordinate analysis (PCoA) (Figure 3A); the weighted (quantitative) UniFrac distance matrix did not show apparent shift between the two groups (Figure 3B). From this result, it could be inferred that the presence/absence of OTUs between the two test groups, OA and RA, was more useful for showing the distance than the abundances of existing taxa. 

### 3.3. Relative Abundances of Bacterial Taxa at Different Taxonomic Levels Between RA and OA Patients

**Figure 4:**
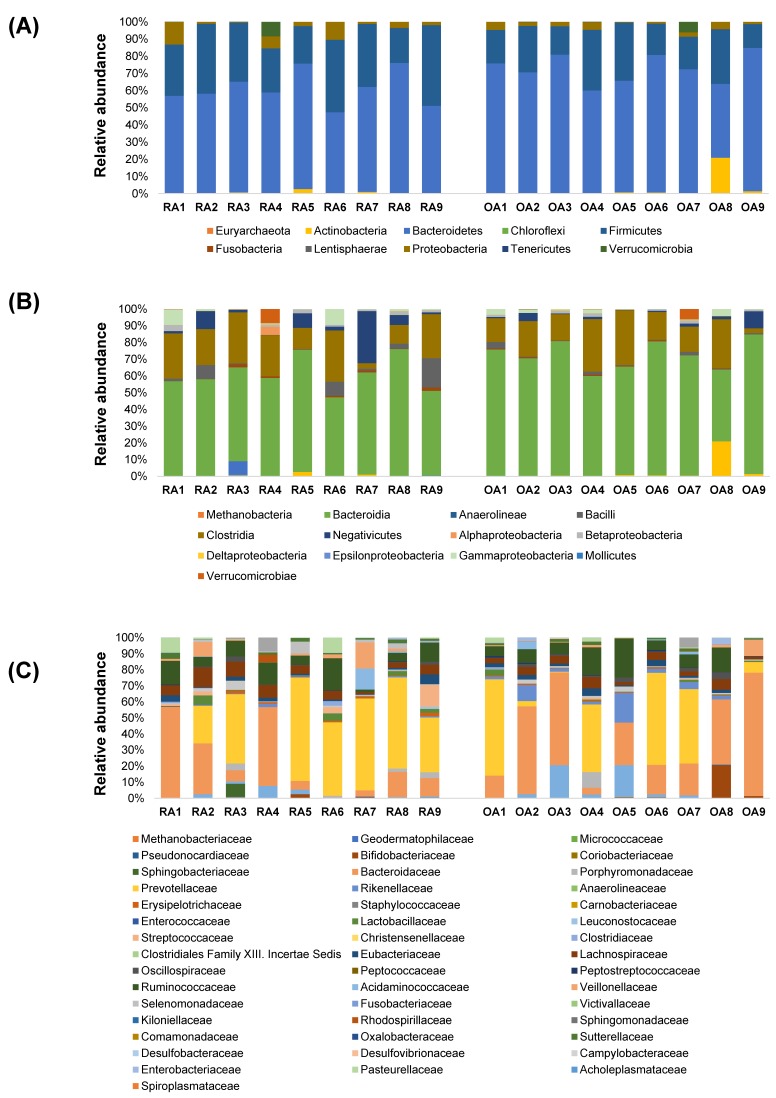
Stacked bar graphs for the relative abundance of bacterial 16S rRNA from fecal samples of rheumatoid arthritis (RA1–9) and osteoarthritis (OA1–9) patients at the (**A**) phylum, (**B**) class, and (**C**) family levels.

**Figure 5:**
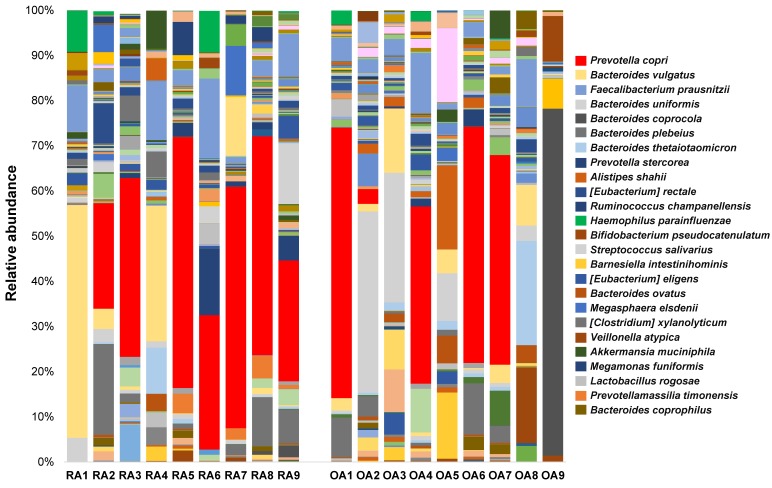
Stacked bar graphs for the relative abundance of bacterial 16S rRNA from fecal samples of rheumatoid arthritis (RA1–9) and osteoarthritis (OA1–9) patients at the species level. Legend on the right side represents the 25 most abundant species arranged chronologically.

The relative abundance of the OTUs, classified as phylum, class, family, and species levels, for each individual sample in the RA and OA groups are shown in the stacked bars in Figure 4 and Figure 5. At the phylum level, there was a noticeable increase in Bacteroidetes and a decrease in Firmicutes in the OA group rather than in the RA group (Figure 4A). At the class level, the presence of Bacilli was more obvious in the RA group than in the OA group; specifically, Bacteroidia were more abundant in the OA group (Figure 4B). At the family level, both groups showed a high abundance of Bacteroidaceae and Prevotellaceae (Figure 4C). 

At the species level, *Prevotella copri* (shown in red color) was the dominant species in most samples from both groups. In the RA and OA groups, seven out of nine samples and four out of nine samples were dominated by *P. copri*, respectively (Figure 5). 

**Figure 6:**
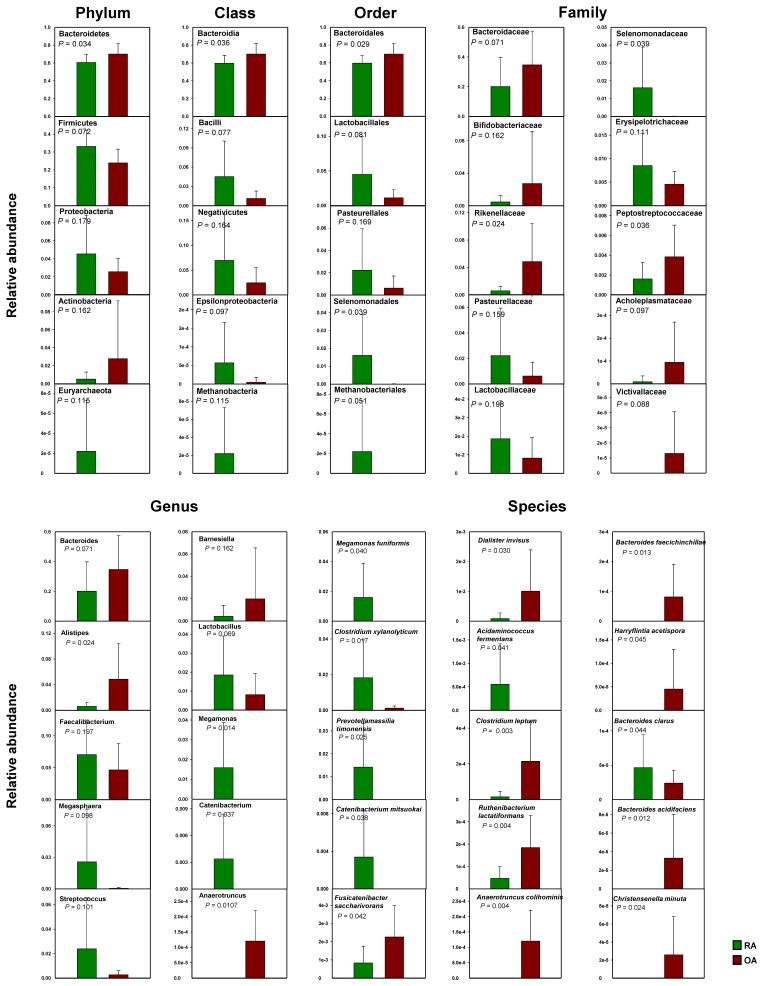
Relative abundance of bacterial taxa varied in the fecal sample from rheumatoid arthritis (RA) and osteoarthritis (OA) patients at the phylum, class, order, family, genus, and species levels. The presented *p*-value was based on independent samples *t*-test (*n* = 9) for each group.

To further investigate the differences in the relative abundance of different bacterial taxa between the RA and OA groups, comparative analyses of the mean relative abundances of the two groups were performed at all taxonomic levels (Figure 6). Adding to the above-mentioned observations, the statistical analyses showed significant differences in the relative abundance of Bacteroidetes between the two groups at the phylum level, with an average of 60.65% and 69.98% for RA and OA, respectively. The relatively rare phylum, Euryarchaeota, was only present in samples from the RA group, but not in the OA group.

At the class level, Bacteroidia were significantly more abundant in the OA group. However, although not statistically significant, the abundance of Bacilli and Negativicutes, was higher in the RA group, at an average of 4.52% and 6.96% when compared with 1.13% and 2.44%, respectively, in the OA group.

At the order level, Bacteroidales were significantly higher in the OA group, while Selenomonadales and Methanobacteriales were only present in the RA group. The relative abundance of Lactobacillales was also higher, although not statistically significant (*p* = 0.081) in the RA group, with an average of 4.51% relative to 1.13% for the OA group.

At the family level, Rikenellaceae and Peptostreptococcaceae were significantly higher in the OA group, while Selenomonadaceae was significantly higher in the RA group. The other important families, Prevotellaceae and Bifidobacteriaceae, were also differentially represented between both groups, although the difference was not statistically significant. The OA group had a higher abundance of Bifidobacterium with an average of 2.72%, while the RA group had an average of 0.047%. In contrast, the Provetellaceae family was more abundant in the RA group, with an average of 36.03% relative to 24% for the OA group.

At the genus level, *Alistipes* and *Anaerotruncus* were significantly higher in the OA group, while *Megamonas* and *Catenibacterium* were significantly higher in the RA group. Other genera that were represented differentially between the two groups included *Megasphaera* (RA, 2.57% and OA, 0.04%), *Streptococcus* (RA, 2.39% and OA, 0.26%), *Lactobacillus* (RA, 1.82% and OA, 0.82%), and *Prevotella* (RA, 33.97% and OA, 23.75%). *Bacteroides* (RA, 20.12% and OA, 34.69%) and *Bifidobacterium* (RA, 0.47% and OA, 2.72%) were higher in the OA group.

At the species level, *Fusicatenibacter saccharivorans, Dialister invisus, Clostridium leptum, Ruthenibacterium lactatiformans, Anaerotruncus colihominis, Bacteroides faecichinchillae, Harryflintia acetispora, Bacteroides acidifaciens*, and *Christensenella minuta* were significantly higher in the OA group. *Megamonas funiformis, Clostridium xylanolyticum, Prevotellamassilia timonensis, Catenibacterium mitsuokai, Acidaminococcus fermentans*, and *Bacteroides clarus* were significantly higher in the RA group (Figure 6). The average relative abundance of *Prevotella copri* was higher in the RA group (30.77%) than in the OA group (22.33%), although the difference was not statistically significant.

**Figure 7:**
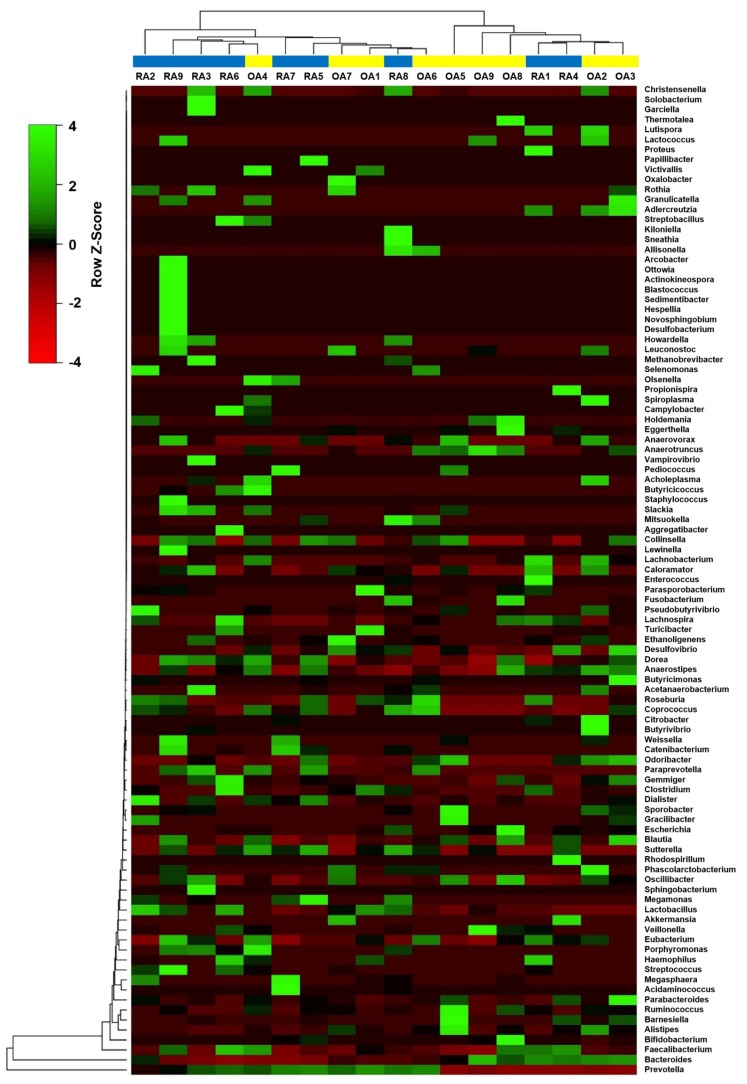
Heatmap of the average linkage hierarchical clustering, based on the Euclidean distance measurements of bacterial genera obtained from nine samples from the RA and OA groups.

The heatmap in Figure 7 shows the average linkage hierarchical clustering, based on the Euclidean distance measurements, of bacterial genera obtained from nine samples from the RA and OA groups. Although there was no distinct cluster in either group, the two main clusters provided insights into the distribution of different genera between the groups. The main distinction was the separation of one cluster with high *Prevotella* abundance from another cluster with low *Prevotella* abundance. The *Prevotella* high abundance cluster contained seven RA samples and four OA samples. Samples in this cluster also had a higher relative abundance of other genera such as *Megamonas, Megashaera, Lactobacillus*, and *Streptococcus*. The *Prevotella* low abundance cluster included samples with a high abundance of *Bacteroides* and other genera, such as *Faecalibacter*, *Barseniella*, and *Parabacteroidetes* (Figure 7).

## 4. Discussion

The gut microbiome may have a significant impact on human health and diseases. A growing body of evidence suggests a link between gut microbiota and several diseases, including autoimmune diseases such as RA. Therefore, characterization of the gut microbiota composition is important for better understanding the association with diseases. Most studies were conducted to compare the gut microbiota composition of diseased versus healthy individuals, and dysbiosis in gut microbiota was often associated with the RA, as reported in several previous studies [7,11,25]. In this study, we compared the gut microbiota of RA and OA patients using 16S rRNA analysis. The analysis of diversity revealed no differences in the complexity of samples. At the phylum level, RA had a lower Bacteroidetes:Firmicutes (B:F) ratio when compared with OA. Rogier et al. [25] reported a decrease in Bacteroidaceae and an increase in Firmicutes during the immune-priming phase of arthritis in the collagen-induced arthritis (CIA) mice model. The study suggested that alteration of the gut microbiome during the immune priming phase could induce an inflammatory response in joints. In a study by Scher et al., a group of patients with new onset RA showed a lack of gut *Bacteroides* when compared with a healthy control group [26]. 

In our study, Bacilli were predominant in RA patients rather than in OA patients. Although it was not significant, *Lactobacillus* was predominant in RA patients. A previous study found that RA could be induced in germ-free animals by exposure to *Lactobacillus* spp., which stimulated T helper type 17 (Th 17) activity and depressed regulatory T cell (Treg) activity [27]. A 2018 fecal microbiota study comparing RA patients and a healthy control group also revealed a significant abundance of Bacilli and Lactobacilli in the RA group [28]. Liu et al. [29] also suggested that the variety and quantity of Lactobacilli was higher in RA patients than in healthy controls. 

In this study, *Prevotella*, especially *P. copri*, were more predominant in the RA group than in the OA group, although this was not a statistically significant result. A previous study by Scher et al. [26] reported a significant expansion of *P. copri* and *Prevotella*-like taxa in patients with new onset RA when compared with a healthy control group. *Bacteroides* and *Bifidobacterium* were decreased in the RA group than in the OA group in this study. Previous studies also showed a decrease in families *Bifidobacterium* and *Bacteroides*, with the addition of genus *Prevotella*, in an early RA group than in the control group [30,31]. Type 1 diabetes mellitus (T1DM), another autoimmune disease, showed a decrease in *Bifidobacterium* when compared with a healthy control group [32]. 

At the species level, *Fusicatenibacter saccharivorans, Dialister invisus*, and *Clostridium leptum* were decreased in the RA group rather than in the OA group in this study. These bacteria were also decreased in other autoimmune diseases, including Crohn’s disease (CD) and ulcerative colitis (UC) [33,34,35]. *Ruthenibacterium lactatiformans* and *Anaerotruncus colihominis* were also decreased in the gut microbiota of RA patients when compared with healthy patients. Another bacterium, *Bacteroides faecichinichillae*, which decreased in RA patients, was also found to decrease in obese people relative to non-obese people. *Bacteroides acidifaciens* was related to insulin sensitivity and thought to be related to T1DM [36]. *Christensenella minuta* showed significantly lower abundance in the RA group in this study and has been previously linked with Parkinson’s disease, the latter being investigated as a possible autoimmune disease [37].

In this study, the lower Bacteroidetes:Firmicutes ratio, reduction in *Bacteroides* and *Bifidobacterium*, and predominance of lactobacilli and *Prevotella* in RA are supported by previous research. At the species level, the gut microbiota (*Fusicatenibacter saccharivorans, Dialister invisus, clostridium leptum, Bacteroides faecichinichillae, Bacteroides acidifaciens,* and *Christensenella minuta*) in RA was decreased when compared with OA. To date, however, there has been no other study that supports the results revealed in this study regarding the RA group. Other researchers have reported that the microbiota mentioned above are linked to other autoimmune diseases.

Several mechanisms have been proposed for the association between gut microbiota and arthritis, including the activation of antigen-presenting cells by affecting Toll-like receptors (TLRs) and Nod-like receptors (NLRs), antigenic mimicry, affecting the intestinal permeability, modulating the host immune system through T cell differentiation, and increase T helper 17 (Th17)-related inflammation [38]. In early studies on murine models, systemic injection with cell walls of certain bacterial species such as *Lactobacillus casei* and *Streptococcus pyogenes*, resulted in polyarthritis [39,40]. Studies involving the use of gnotobiotic mice have shown that gut microbiota dysbiosis can increase the production of pro-inflammatory cytokines, interleukin-17, and Th17 cells, even in tissues rather than the gut [10]. 

The considerable body of evidence on the association between dysbiosis and the action of certain gut microbes with the onset and progress of RA has triggered research on novel therapeutic targets and strategies to enrich and restore healthy gut microbiome as an adjunctive therapy. Previous studies on RA patients have shown that supplementation with probiotics was associated with functional improvement and enhancement of the wellbeing of patients; although there were no significant differences in the clinical characteristics of RA [41,42]. More recently, Vaghef-Mehrabany et al. [43] reported significantly improved disease activity and inflammatory status of RA patients by probiotic supplementation. In another study on murine models, the loss of beneficial bifidobacteria and the abundance of pro-inflammatory microbes were associated with obesity and increased inflammation in obesity OA. Supplementation with the non-digestible prebiotic fiber, Oligofructose, have resulted in restoration of a lean gut microbiome, resulting in reduced inflammation and protection from OA [44]. The beneficial effect of probiotics is mainly related to their antimicrobial effects, enhancement of mucosal barrier integrity, and immune modulation [45]. Further studies are required to confirm the roles of probiotics and prebiotics supplementation and other methods of positive gut microbiome manipulation in combating autoimmune and inflammatory diseases and improving the effectiveness of such methods.

Limitations of this study include the small sample size and absence of healthy controls. Additionally, factors that could affect gut microbiota, such as sex, age, and BMI, were not investigated. Nonetheless, this is the first study to compare the gut microbiota of RA and OA patients. 

## 5. Conclusions

In this study, we found a difference in the gut microbiota of the RA and OA groups. The tendency of RA dysbiosis compared with that of OA was similar to results seen in other autoimmune diseases, indicating that the specific gut microbiome pattern may be linked with autoimmunity. Although the impact of microbiota on OA pathogenesis may be present in conjunction with individual-specific risk factors, as reported recently, effects of gut microbiota on RA pathogenesis, specifically autoimmunity and T cell immunity, were more significant. 

Furthermore, a more accurate analysis of the microbiome’s effect on RA and OA progression may be possible by comparing RA and OA patients with a healthy control group. In addition, it may be helpful to elucidate the pathogenesis and treatment of RA by investigating the linkage between the dysbiosis of gut microbiome and the presence of anti-cyclic citrullinated peptide (CCP) as a biomarker for RA, Th 17, Treg activity, and pro-inflammatory cytokines.

## Figures and Tables

**Table 1:** Characteristics of study participants.

	Rheumatoid Arthritis (*n* = 9)	Osteoarthritis (*n* = 9)	*p* Value
Age	55.00 ± 5.21	59.78 ± 8.18	0.182
Weight	61.47 ± 7.36	58.59 ± 7.77	0.458
BMI	24.66 ± 3.21	24.48 ± 2.86	0.907
ESR (mm/h)	32.33 ± 12.82	18.11 ± 8.17	0.018
hsCRP (mg/dL)	0.46 ± 0.56	0.13 ± 0.25	0.035

BMI, body mass index; ESR, erythrocyte sedimentation rate; hsCRP, high sensitivity C-reactive protein. The presented *p*-value was based on independent samples *t*-test.

## Supplementary Materials

The following are available online at https://www.mdpi.com/2073-4425/10/10/748/s1. Table S1: Characteristics such as disease duration, disease activity, RF, anti-CCP, and medication use among the rheumatoid arthritis (RA) patients in this study.
