# Supplementary material for: Comparative Analysis of Fecal Microbiota Composition Between Rheumatoid Arthritis and Osteoarthritis Patients
Source: Genes (Basel). 2019 Sep 25;10(10):748. doi: 10.3390/genes10100748 (PMC6827100; doi:10.3390/genes10100748)
Supplement: Supplementary file 1 [file genes-10-00748-s001.pdf]

Table S1. Characteristics of rheumatoid arthritis patients in this study

Patient No.	Age (years)	Disease duration (months)	DAS28 -CRP	DAS28 -ESR	RF (IU/mL)	Anti-CCP Ab (U/ml)	Antirheumatic drug use in 3 months			
							MTX	HCQ	SSZ	GC
1	54	12	1.37	2.64	31.9	300.0	Y	Y	N	Y
2	60	50	1.66	2.95	15.3	0.3	Y	N	N	N
3	58	103	2.91	3.63	107	112.5	Y	Y	N	N
4	54	29	1.8	3.12	39.6	41.9	Y	N	Y	Y
5	50	115	2.85	4.67	80.4	100.0	Y	N	N	Y
6	61	31	2.64	4.01	39.1	192.9	Y	N	N	Y
7	44	94	2.91	4.42	5.4	0.5	Y	Y	N	Y
8	60	89	2.63	4.07	5.1	1.7	N	N	Y	N
9	54	35	2.22	4.05	9.3	0.2	Y	N	Y	Y

DAS28, Disease Activity Score with 28-joint assessment; CRP, C-reactive protein; ESR, Erythrocyte Sedimentation Rate; RF, Rheumatoid Factor; Anti-CCP Ab, Anti-Cyclic Citrullinated Peptide Antibody; DM, Diabetes Mellitus; HTN, Hypertension; MTX, Methotrexate; HCQ, Hydroxychloroquine; SSZ, Sulfasalazine; GC, Glucocorticoid; N, No; Y, Yes.
